# Synthetic DNA Vaccines: Improved Vaccine Potency by Electroporation and Co-Delivered Genetic Adjuvants

**DOI:** 10.3389/fimmu.2013.00354

**Published:** 2013-11-04

**Authors:** Seleeke Flingai, Matias Czerwonko, Jonathan Goodman, Sagar B. Kudchodkar, Kar Muthumani, David B. Weiner

**Affiliations:** ^1^Department of Pathology and Laboratory Medicine, Perelman School of Medicine at the University of Pennsylvania, Philadelphia, PA, USA; ^2^Department of Anatomy, School of Biomedical Sciences, Austral University, Pilar, Argentina

**Keywords:** DNA vaccine, plasmid, electroporation, adjuvants, interleukin-12

## Abstract

In recent years, DNA vaccines have undergone a number of technological advancements that have incited renewed interest and heightened promise in the field. Two such improvements are the use of genetically engineered cytokine adjuvants and plasmid delivery via *in vivo* electroporation (EP), the latter of which has been shown to increase antigen delivery by nearly 1000-fold compared to naked DNA plasmid delivery alone. Both strategies, either separately or in combination, have been shown to augment cellular and humoral immune responses in not only mice, but also in large animal models. These promising results, coupled with recent clinical trials that have shown enhanced immune responses in humans, highlight the bright prospects for DNA vaccines to address many human diseases.

## Introduction

Prevention is the most foolproof method of medical intervention, and the vaccine is its most representative example. Since Edward Jenner’s pioneering smallpox vaccine, vaccinology has followed an irregular path to its modern day form, with alternating periods of progress and stagnation (1, 2). Through advances in molecular biology, vaccinology has evolved from using basic inoculations of whole microorganisms to harnessing the power and flexibility of genetic engineering (3). DNA vaccination, one of the latest biotechnological breakthroughs, is the beginning of a new chapter in vaccine technology.

The fundamental idea behind DNA vaccines (also known as genetic vaccines) is to induce immune responses against recombinant antigens encoded by genetically engineered DNA plasmids expressed *in vivo*. After immunization, host cellular machinery facilitates the expression of plasmid-encoded genes, which leads to the generation of foreign antigens that can be processed and presented on both major histocompatibility complex (MHC) class I and class II molecules. These host-synthesized foreign antigens can be recognized by the immune system, inducing a complete and effective immunization.

This novel method of vaccination was engineered in response to a series of emerging diseases that remain without proper prophylactic and therapeutic treatment. More than 50 years ago, pioneering studies carried out by Atanasiu et al. and Orth et al. showed that inoculation of mouse-derived tumor DNA induced tumors and led to seroconversion in injected mice (4, 5). The work of Wolff et al. showed that DNA plasmids injected intramuscularly (i.m.) could generate long-term gene expression *in vivo* without the need for a special delivery system (6); this finding helped generate much excitement for the scientific community. Within the past decade, four successful DNA plasmid products have been licensed for animal use: one for the treatment of West Nile virus in horses (7), one against hematopoietic necrosis virus in salmon (8), one for the treatment of melanoma in dogs (9), and a growth hormone-releasing hormone (GHRH) gene therapy for swine (10). However, despite promising studies in small animal models and improved efficacy in large animal models, the clinical ability of DNA vaccines still remains unproven. While the reasons for this inconsistency have yet to be fully elucidated, several attempts have been made to enhance immunogenicity in humans, resulting in studies that have provided a wealth of constructive information that may guide research efforts toward the development of improved DNA products.

This review will focus on specific combined DNA vaccine approaches to improving immunogenicity in humans. In particular, we highlight *in vivo* electroporation (EP) and the use of genetically encoded immune adjuvants. These important technological advancements have helped drive the field of DNA vaccines into a modern resurgence, and the use of these techniques – along with improved protocols and methods for synthetic gene production – may be the key to successfully controlling a number of human diseases.

## Beginnings of DNA Vaccination

The seeds of DNA vaccinology were planted in the mid-twentieth century, when studies by Stasney et al. (11), Paschkis et al. (12), and Ito (13) demonstrated the ability to transfer DNA to animal cells by injection of crude DNA preparations isolated from tumors. These reports and others laid the groundwork for DNA vaccines by showing that DNA injection into animals can result in the expression of the delivered genes *in vivo*. However, perhaps the most important aspect of these early studies was that the immune system could respond to the gene products generated by DNA inoculation. For example, Atanasiu et al. (4) and Orth et al. (5) purified DNA extracts from polyoma viruses and demonstrated both tumor induction and the generation of anti-polyoma antibodies in injected animals. These findings were extended in studies by Israel and colleagues, who observed that injection of recombinant purified polyoma virus DNA resulted in tumor formation and anti-polyoma antibody production (14). Will and coworkers also observed humoral immune responses after inoculation of recombinant purified hepatitis B viral DNA into chimpanzees (15).

While many of these initial studies primarily focused on studying viral DNA biology (with humoral immunity against the inoculated gene product being of secondary importance), later studies sought to specifically study plasmid gene expression *in vivo* for a variety of applications. For example, Benvenisty and Reshef delivered genes encoding insulin and human growth hormone (HGH) into newborn rats, resulting in their expression *in vivo* (16). Later, studies by Jon Wolff and colleagues demonstrated long-term expression of DNA plasmids injected intramuscularly in mice (6). And in 1992, Tang et al. directly studied the immune response in mice elicited by DNA inoculation of foreign proteins. Using a gene gun to shoot gold particles coated with HGH-encoding DNA into mouse skin, the researchers found detectable levels of antibodies against the hormone, thus reproducing the earlier work of Israel, Atanasiu and Orth but in a more controlled manner (17). At the annual Cold Spring Harbor Vaccine meeting in September 1992, the laboratories of Margaret Liu (Merck), Harriet Robinson (University of Massachusetts), and David Weiner (University of Pennsylvania) independently reported that plasmid delivery into small animals could induce antibodies and cytotoxic T lymphocytes (CTLs) against influenza virus (18, 19) or HIV (20). Together, these studies were instrumental in laying the groundwork for the DNA vaccine field.

To date, three DNA vaccines and one DNA-based hormone therapy have been licensed for veterinary use, illustrating the advancements in DNA plasmid technology that have allowed these products to be successful in animals both big and small. A DNA vaccine for West Nile Virus in horses, licensed in 2005, was first shown to be efficacious in mice and horses before licensure (7). After licensure, the vaccine entered phase 1 clinical trials and was shown to induce neutralizing antibodies in healthy adults (21, 22). Also licensed in 2005, the infectious hematopoietic necrosis virus vaccine targets school salmon and has resulted in improved food quality and quantity (8, 23, 24). The canine melanoma vaccine, fully licensed in 2010 after conditional licensing in 2007, encodes human tyrosinase, allowing the immune system to break tolerance to canine tyrosinase and generate an effective immune response against tumor cells (9, 25). Another groundbreaking use of DNA plasmids that received licensure in 2007 was the GHRH product for use in swine (10). As the first licensed EP-delivered product and the first licensed gene therapy product, DNA-encoded GHRH, which causes an increase in growth hormone, has allowed more piglets to be weaned by improving maternal performance (26). All of these breakthroughs, some of which have taken place in animals larger than humans, have validated the very real potential of DNA product licensure for human use.

## DNA Vaccine Mechanism: How Does It Work?

DNA vaccination is an attractive immunization platform due to its ability to elicit potent CTL responses while preserving the capacity to stimulate other arms of the immune system. DNA vaccines achieve this goal by mimicking aspects of natural viral infections: the expression of foreign genes delivered *in vivo* results in the production of proteins that are processed and presented to the immune system quite similarly to proteins encoded by viral genes. The end result of DNA vaccination is the production of non-live, non-replicating, non-spreading antigens that can induce not only CD4^+^ and CD8^+^ T-cell immunity, but also B-cell immunity. The details of the mechanisms by which some components of DNA vaccination are achieved is still under investigation. Nonetheless, many of the key steps of DNA immunization have been partially elucidated.

Two major cell types are required for initiating the immune response elicited by DNA vaccination: somatic cells (primarily myocytes or keratinocytes) and professional antigen-presenting cells (APCs). For example, after intramuscular injection of a plasmid, myocytes, resident dendritic cells (DCs), and monocytes are transfected (27–30). Upon entering the nucleus of transfected cells, the plasmid-encoded genes are expressed, and foreign antigens are generated and processed into peptide strings by host cell machinery. These peptides can then be associated with the MHC class I or II molecules of APCs, affording these cells the ability to prime naïve T cells in the draining lymph nodes. While direct transfection of DCs is one method in which vaccine-derived endogenous peptides can form complexes with MHC class I molecules and prime naïve CD8+ T cells, DCs may also cross-present cell-associated exogenous antigen obtained from engulfing apoptotic or necrotic transfected cells (31, 32). DCs may also display peptides via MHC class II molecules by capturing antigen secreted from transfected cells. Ultimately, plasmid products are capable of accessing both pathways, resembling many aspects of viral protein immune induction.

## Advantages of DNA Vaccines

Since its inception, there has been great promise in DNA vaccination. Using genetic material as a vector for immunization offers a number of advantages over traditional vaccine modalities in terms of effectiveness, safety, and cost. For years, scientists have put great effort into maximizing these strengths in order to establish DNA vaccines as a central component of preventative and therapeutic medicine.

### Immunological advantages

The main advantage of DNA vaccines is their ability to stimulate both the humoral and cellular arms of the adaptive immune system. In regards to humoral immunity, the generation of antibodies by B lymphocytes against invading pathogens is one of the most effective defenses mounted by the immune system. Vaccines that utilize live-attenuated microorganisms, killed viral particles, or recombinant viral proteins elicit the production of specific antibodies that bind superficial microbial structures on the target pathogen. Unfortunately, immunological pressure or imprecise genome replication can cause certain pathogens to accumulate mutations that reduces the effectiveness of antibodies originally generated against the pathogen. Typically, antibody responses generated by traditional vaccines target only the specific antigens found in the inoculum, and are poorly able to control similar pathogens that carry either subtle or gross changes to the antigen. Due to the ability to genetically modify the antigen encoded by DNA vaccines, the vaccine can be designed to contain the most highly conserved regions of the superficial, antibody-generating structures on a pathogen, providing a means to generate broadly neutralizing antibodies against pathogens such as HIV and the influenza virus.

Regarding cellular immunity, CTLs eradicate infected or malignant cells upon recognition of foreign antigens in complex with MHC class I molecules on the target cell. Live-attenuated microorganisms can enter cells, and their viral proteins can be processed and directed to the MHC class I pathway for presentation upon the cell surface and the subsequent induction of CTL-mediated adaptive immunity. DNA vaccines also enter cells and produce antigen that can be processed and presented via MHC class I; however, DNA vaccines eschew the reversion risks associated with live-attenuated microorganisms.

Another major advantage of the DNA vaccine model is its versatility. In addition to the prevention of infectious diseases, DNA vaccines may also be used to treat malignancies and autoimmune or genetic disorders. When used for cancer therapy, plasmid DNA encoding a tumor-associated antigen (TAA) can be designed to induce CTL responses against cancerous cells expressing the antigen (33). Concerning autoimmune disorders, DNA plasmids may encode immunomodulatory proteins that could tailor the immune response to the type and intensity needed to ameliorate conditions as common as juvenile diabetes or food allergies.

### Safety advantages

Vaccines as a whole have maintained a very strong safety profile. Nevertheless, live-attenuated and inactivated pathogens used in traditional vaccines carry the potential to return to virulence, which may cause pathogenic infections *in vivo* (34, 35), particularly in immunocompromised individuals. DNA vaccines, on the other hand, do not use microorganisms and therefore avoid the risk of reversion. Additionally, frequent vaccine-induced side effects such as headache, fever, and transient pain have shown reduced rates with DNA vaccines (36). Investigations into the possibility of DNA vaccine plasmids integrating into the host chromosome have not shown relevant levels of integration to occur (37, 38). Furthermore, preclinical and clinical studies have not detected detrimental anti-vector autoimmunity (i.e., disease-causing anti-nuclear or anti-DNA antibodies) after DNA vaccination, making it possible to administer multiple doses of DNA vaccines without triggering an immune reaction to the plasmid vector (39, 40); such an immunization protocol may be particularly useful for therapeutic cancer vaccination, which relies on repeated boosting of T-cell responses to be effective. This is in contrast with viral or bacterial vectors, which often induce anti-vector immunity that prevents boosting with the same vector (41). Lastly, while there has been evidence of anti-DNA antibodies generated as a result of epitope spreading (42), these antibodies were found to be transient and, most importantly, purely innocuous in animal models (43).

## DNA Vaccine Enhancements: Improving Immunogenicity

### DNA construct optimization

Early in the development of DNA vaccines, it became clear that maximizing the expression and synthesis of the encoded antigen was vital to the induction of strong and potentially protective immune responses. Bolstered by technological advancements in the DNA vaccine field, the relative simplicity of naked plasmid DNA has gradually given rise to a series of more sophisticated products that confer higher levels of immunogenicity to DNA vaccines.

Eukaryotic promoters, for instance, are no longer used, and most DNA vaccines now rely on a strong viral promoter for optimal transgene expression levels. Popular promoters taken from human oncogenic viruses such as simian virus 40 (SV40) (44) or Rous sarcoma virus (RSV) (45) have been replaced by effective non-carcinogenic alternatives: the human cytomegalovirus immediate early promoter (hCMV-IE) (46) and the CMV-chicken-β-actin (CAG) promoter have been shown to induce high constitutive expression in a wide range of mammalian cells (47), and are now the most widely used promoters in current preclinical studies and clinical trials. However, the choice of a promoter is still a delicate matter; some studies have demonstrated that the inherent strength of viral promoters does not necessarily correlate with DNA vaccine efficacy *in vivo*, partly due to various cytokines attenuating viral promoter activity (48). IFN-γ, one of the primary proteins responsible for this effect, is secreted to inhibit the propagation of viruses by inducing transcriptional repressors that downregulate viral replication (49). Consequently, both cellular (50, 51) and hybrid (52) promoters are currently being tested as possible alternatives to viral promoters. The human MHC class II promoter has been shown to be a weak but interesting alternative, particularly because the protein controlling its expression – the MHC class II transactivator (CIITA) – is upregulated by antiviral cytokines (53).

In addition to viral promoters, transcriptional transactivators and other enhancer elements can also increase transcription activity. Most of the transactivator genes that have been evaluated thus far have viral origins, such as the regulatory R region from the 5′ long terminal repeat (LTR) of human T-cell leukemia virus type 1 (HTLV-1). This particular element, combined with a CMV promoter, has been shown to induce a suggestively higher cellular immune response to HIV-1 compared to the analogous parental DNA vaccines in both mice and non-human primates (54). Despite promising results, the use of such regulatory enhancers is of some concern for off target effects and will need additional investigation.

Improving vaccine potency by optimizing translation efficiency is also an active area of research. The insertion of a Kozak sequence flanking the AUG initiator codon (ACCAUGG) within mRNA may facilitate its recognition by eukaryotic ribosomes (55). Several studies have demonstrated that the presence of a Kozak sequence adjacent to the start codon has a positive influence on gene expression from DNA plasmids (55, 56). Moreover, proper termination is as important as proper initiation; double stop-codons can be added to prevent read through, which could lead to oversized and/or misfolded proteins (57).

Codon optimization is another common and highly efficient technique used to enhance protein production (58). Not all organisms use certain codons equally; this is due to variable levels of transfer RNAs (tRNAs) within cells, resulting in a usage preference for certain codons between species. As a result, unmodified bacterial or viral genes may not be optimally translated in eukaryotic cells. Therefore, designing DNA plasmid constructs in which the codon usage is optimized for eukaryotic cells may lead to more efficient translation, resulting in enhanced protein production (59). Consequently, augmented antigen expression can enhance both humoral and cellular responses (59–66). By using similar technology, it is also possible to add sequences that improve stability and thus translation of mRNA, such as leader sequences and polyadenylation signal sites; conversely, removing elements that decrease stability – such as secondary mRNA structures that inhibit ribosomal loading or cryptic sequences that inhibit mRNA nuclear export – can also improve mRNA stability and translation.

Lastly, there are modifications that do not directly affect the transcription or translation, but instead cause changes in the constitution or destination of the final protein. Protein modifications that enable cell surface expression or secretion (e.g., inclusion of secretion signal sequences) are commonly linked with augmented immunogenicity (67, 68). Targeting the expressed protein to specific intracellular pathways such as the proteasomal pathway may also increase MHC class I-restricted presentation. Additionally, current technology offers the possibility of finding conserved and common sequences among different pathogens (also called consensus sequences). Using these sequences as transgenes for immunization may maximize protection against multiple and highly variable pathogens (68–70).

### Electroporation

Of the many advancements in DNA vaccines that have drastically improved immunogenicity, plasmid delivery via *in vivo* EP has proven to be one of the more impactful enhancements. EP involves the application of brief electric pulses to the vaccination site after injection of plasmid DNA. Administering EP results in the formation of transient pores in the plasma membrane of cells at the injection site (71, 72), which allows macromolecules such as nucleic acids to enter the cytoplasm (73). While the mechanisms for plasmid delivery by EP are still incompletely understood, the procedure improves plasmid delivery by a factor of 10–1,000 fold over naked DNA delivery alone (74). After the cessation of the electric pulses, pore closure traps the macromolecules within the cytoplasm. Not only does EP mediate enhanced plasmid uptake, but it also increases DNA distribution throughout the tissue and causes a local inflammatory reaction, both of which contribute to a stronger immune response (75). Importantly, the safety profile of EP after DNA vaccination is very similar to that of DNA delivered without EP, with no increased risk of toxicity or integration of the DNA plasmid into transfected cells. The most common adverse event described in clinical trials involving DNA vaccination with EP was increased pain at the application site.

**Figure 1:**
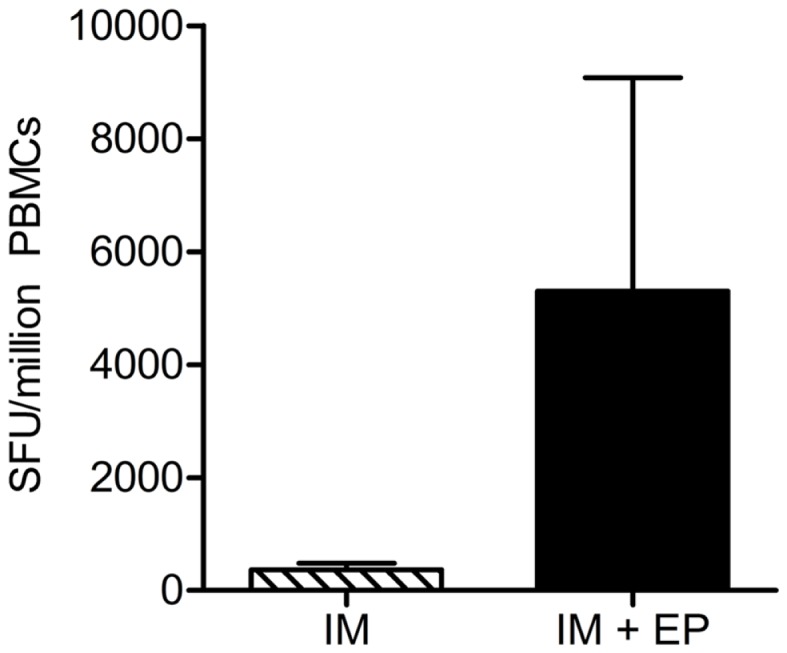
**Delivery of DNA vaccine with *in vivo* electroporation enhances cellular immune responses**. A DNA vaccine encoding HIV-1 consensus immunogens was injected intramuscularly with or without EP into rhesus macaques. Interferon (IFN) gamma ELISpots were performed 2 weeks after the third (final) immunization; total antigen-specific cellular responses are shown, *n* = 5 per group. ELISpot, enzyme-linked immunospot assay; HIV-1, human immunodeficiency virus-1; SFU, spot-forming units; PBMCs, peripheral blood mononuclear cells; IM, intramuscular injection; IL, interleukin. Modified from Hirao et al. (77).

While directly translating enhanced plasmid delivery to improved gene expression and immune responses is not without difficulty, comparison studies using reporter gene systems or immunogenicity readouts have established a strong correlation between EP delivery and augmented gene expression and immune responses (Figure 1) (30, 76, 77). Furthermore, these improvements in DNA vaccine expression and potency can be achieved at significantly lower doses than with naked DNA delivery alone. A number of preclinical studies in small and large animal models have generated a substantial profile on the application of EP with DNA vaccination. For example, administration of the HIV DNA vaccine ADVAX was shown to increase antigen-specific CD4^+^ and CD8^+^ T-cell responses in mice when delivered by EP (78). Additional preclinical studies in pigs (79), cows (80, 81), rabbits (82), and others have had similar positive results in their respective DNA vaccine models with EP. A recent study compared protective antibody responses in chickens given a DNA vaccine containing the hemagglutinin (HA) gene of the avian influenza H5N1 virus delivered with or without EP (83): of the chickens that had the vaccine delivered by EP, 100% showed complete protection (low viral load and absence of clinical symptoms and mortality), while only 20% of the chickens who received the vaccine without EP developed antibodies.

However, the largest impact of EP on DNA vaccination has come from its promising effects in large animals such as non-human primates. When an optimized, synthetically developed SIV DNA vaccine was delivered by adaptive EP to rhesus macaques, the vaccine induced a greater magnitude of IFNγ-producing cells, greater proliferative capacity of CD8^+^ T cells, and increased polyfunctionality of CD4^+^ and CD8^+^ T cells compared to the Merck adenovirus serotype 5 (Ad5) SIV vaccine (84). This important study showed that DNA vaccines had indeed made long strides toward improving immunogenicity in large animals, surpassing the potency of the live vector Ad5. Numerous studies in non-human primates – using DNA vaccines for diseases such as anthrax (85), monkeypox (86), and malaria (87, 88) – have further emphasized the impact of EP on drastically enhancing immunogenicity in large animals.

The augmented immunogenicity observed in preclinical studies has also carried over to clinical trials. Recent results from a human papillomavirus (HPV) 16/18 DNA vaccine phase I trial have shown that vaccination with adaptive EP induced HPV-specific CD8^+^ T cells that exhibited robust cytolytic functionality (89). Furthermore, almost all the vaccinated women in this study seroconverted with high titer to the antigens in the vaccine. The immune response induced by the DNA vaccine was superior to both viral and non-viral vaccines previously tested by others in the same disease model (90–94). In a phase I trial of a therapeutic approach for an HIV DNA vaccine ADVAX, static EP delivery of the vaccine elicited an improved HIV-specific cell-mediated immune response compared to vaccination without EP (95). However, there was no difference in antibody levels between the two delivery methods. Furthermore, DNA vaccination with EP delivery has been shown to induce humoral responses following administration of a prostate cancer DNA vaccine with EP (96). These results illustrate the immense progress DNA vaccination has made over the past decade, with the induction of strong responses that may prove beneficial against the diseases targeted.

As with any technology in its early stages of development, additional work needs to be done to optimize EP in order to modulate the immunogenicity of DNA vaccines and reduce the associated side effects – namely, the pain generated at the application site. Alteration of the pulse patterns, electrode configurations, impedance of target tissues, and additional factors all can influence the immune response elicited by the DNA vaccine. By employing different types of electrodes, EP can be compatible with both i.m. and i.d. delivered DNA vaccines (76, 97–100) and can also be used in conjunction with chemical formulations or other mechanical approaches for better results. For example, *in vivo* EP of porcine skin after injection of plasmid in combination with aurintricarboxylic acid (ATA) was shown to increase transgene expression ∼115-fold relative to plasmid injection alone, 2- to 3-fold over DNA with EP, and 17-fold over DNA combined with ATA (101). In the same manner, a microneedle array with electrical functionality has shown encouraging results in human epidermal cells as well as human red blood cells (102). Recent optimizations to a minimally invasive surface intradermal EP device have shown that low-voltage EP applied to the skin can elicit robust humoral and cellular immune responses without tissue damage (103). Some of these changes to the EP protocol may be broadly applicable to a number of different DNA vaccines, while other DNA vaccines will require specialized tweaks to the EP protocol to generate the precise immune response needed to combat the intended target.

### Genetic enhancing strategies: adjuvants

Because low immunogenicity has been the major deterrent toward using DNA vaccines in large animals and humans, several approaches have been investigated to increase the intensity and duration of vaccine-induced immune responses. One popular strategy has been to create vaccine cocktails, which includes the DNA vaccine along with plasmids encoding immunomodulatory proteins. Such adjuvant-encoding genes can be delivered either as separate plasmids or as additional genes encoded by the antigen-encoding plasmid. Upon vaccination, cells transfected with the adjuvant-encoding plasmid can express and secrete the molecular adjuvant into the surrounding region, affecting local APCs and cells in the draining lymph node. The end result is long-lasting, low level production of immunomodulatory cytokines that can tailor the immune response to the demands of each particular pathogen. For example, protection from certain viruses, other intracellular pathogens, or tumors may benefit from the use of cytokines that induce Th1-type immunity, such as IL-2, IL-12, IL-18, and IFNγ, which all generally promote cell-mediated immune responses (104). Conversely, cytokines such as IL-4 and IL-5 may be useful against extracellular pathogens while IL-10 and transforming growth factor-β (TGFβ) may prove effective in treating autoimmune disorders that arise from aberrant cell-mediated immunity (104). And while the role of Th17 cells during infection varies from pathogen to pathogen, evidence suggests that this cell subtype assists in the resistance to a number of bacterial and parasitic infections such as *Leishmania* (105, 106), *Pseudomonas aeruginosa* (107), *Mycobacterium tuberculosis* (108), and others. Cytokines such as TGFβ and either IL-6 or IL-21 are required for Th17 differentiation and may be useful for directing a Th17-type immune response during vaccination. By raising the concentration of certain immunomodulatory proteins during the initiation or boosting of an immune response, one can selectively activate or inhibit the division of the immune system that would lead to the greatest immunological benefit.

Another adjuvant strategy involves using plasmids encoding cytokines capable of recruiting, activating, and/or enhancing the activity of APCs. Granulocyte-macrophage colony-stimulating factor (GM-CSF), a white blood cell growth factor, is perhaps the best characterized example of this adjuvant approach. Plasmid-encoded GM-CSF, when used in combination with a rabies virus DNA vaccine, was shown in mice to increase CD4^+^ T-cell responses, antibody production, and protection from lethal challenge (109). Similarly promising results have been seen in murine models of HIV (110, 111), herpes simplex virus-2 (HSV-2) (112), encephalomyocarditis virus (113), and hepatitis C virus (114). Unfortunately, these results were not recapitulated in clinical trials, allegedly due to a relative lack of GM-CSF receptors on human APCs compared to their murine counterparts (115). Nonetheless, other cytokines are currently being studied as candidate DNA vaccine adjuvants. CXC chemokines such as IL-8 (116), as well as CC chemokines such as macrophage inflammatory protein (MIP)-1α (117, 118), MIP-3α (118), MIP-3β (119), and RANTES (120), may increase the potency of the immune response. Ultimately, these proteins can augment vaccine-induced immune responses by coordinating the movement and functionality of leukocytes important for antigen presentation; this suggests that the immunogenicity of DNA vaccines may be limited by the availability of APCs at the site of inoculation.

DNA vaccine immunogenicity can also be enhanced by co-delivering plasmid-encoded co-stimulatory and adhesion molecules. For example, administration of B7-1 (CD80) and B7-2 (CD86), proteins that provide the crucial second signal required for T-cell activation (121, 122), has been shown to increase CTL activity in cancer and HIV, respectively (123–125). Additionally, blockade of co-inhibitory molecules has been another strategy employed in recent DNA vaccination research. For example, blocking interactions between the co-inhibitory receptor programed death 1 (PD-1) on T cells with its ligands PD-L1 or PD-L2 has been shown to prevent negative regulation of T-cell responses during chronic viral infection and cancer (126). Plasmid-encoded soluble PD-1, when used in combination with either HPV (127) or HIV (128) DNA vaccines, was found to increase antigen-specific CD8^+^ T-cell responses compared to the vaccine alone in mice. In the case of adhesion molecules, interactions between the ligands LFA-3 and ICAM-1 (expressed on APCs) and their corresponding receptors CD2 and LFA1, respectively (expressed on T cells) facilitate the formation of a stable cellular synapse and contribute to optimal signaling between cells. Kim et al. demonstrated that the co-injection of the genes for these adhesion molecules – particularly LFA-3 – together with plasmid DNA leads to an increase in antigen-specific lymphoproliferative and cytotoxic responses (111). Importantly, while the options for gene-based immunomodulatory proteins are almost overwhelmingly numerous, the real challenge resides in finding the combination and timing suitable for each adjuvant that will lead to protective results in clinical trials.

### Interleukin-12

**Figure 2:**
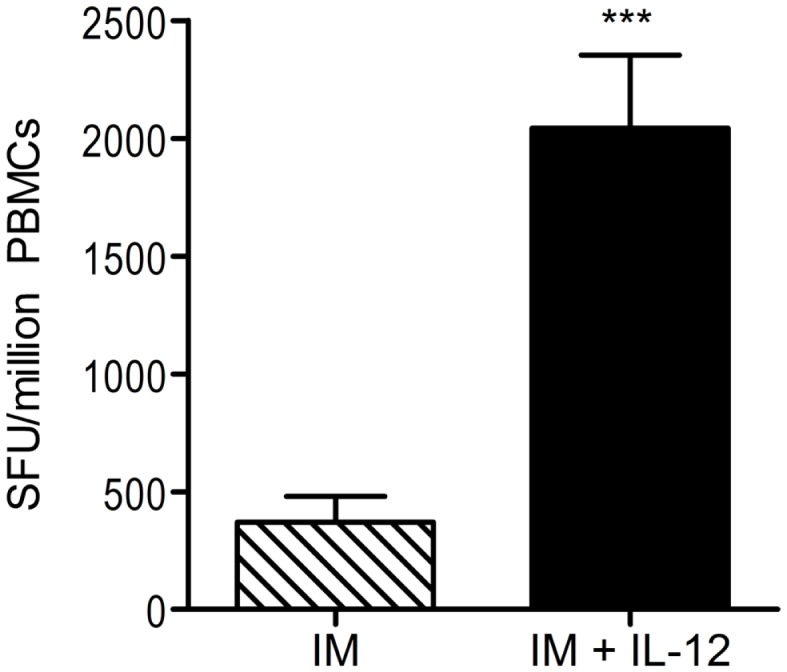
**Co-administration of DNA vaccine with plasmid IL-12 increases cellular immune responses**. A DNA vaccine encoding HIV-1 consensus immunogens was administered intramuscularly (without EP) to rhesus macaques with or without plasmid-encoded IL-12. Interferon (IFN) gamma ELISpots were performed 2 weeks after the third (final) immunization; total antigen-specific cellular responses are shown. *n* = 5 per group; ***represents *p* < 0.001. ELISpot, enzyme-linked immunospot assay; HIV-1, human immunodeficiency virus-1; SFU, spot-forming units; IL, interleukin. Modified from Hirao et al. (77).

One particular cytokine that has received extensive attention in the DNA vaccine field is interleukin-12 (IL-12). IL-12 plays a key role in adaptive immunity as a driving force in T-helper cell type 1 immunity; as such, it has been shown to stimulate the production of IFNγ by T cells and augment CD8^+^ cytotoxic T-cell activity (129). As one of the earliest Th1-specific cytokine genetic adjuvants explored (130), work from our laboratory and collaborators have revealed the strong activity of IL-12 as a DNA vaccine adjuvant (Figure 2). For example, co-delivery of plasmid IL-12 increases the activation, proliferation, and effector function of T cells in NHP (131, 132). This increase in functional T cells includes a 22-fold improvement in CTL responses and enhanced protection in a macaque challenge model. Additional studies in large animals have further highlighted the strong adjuvant effects of plasmid IL-12 (133–137). Additionally, delivering an HIV DNA vaccine with plasmid IL-12 by EP in macaques resulted in an integrative increase in the magnitude and proliferative capacity of antigen-specific IFNγ-producing cells compared to i.m. DNA immunization alone (77). Preclinical studies in other disease models such as hepatitis C (138), HSV-2 (139), and *Toxoplasma gondii* infection (140) further illustrate why plasmid-encoded IL-12 is a fantastic molecular adjuvant for DNA vaccination.

These important preclinical studies have led to exploring IL-12 as a genetic adjuvant in human clinical trials. First-in-human synthetic HIV DNA vaccine trials in combination with IL-12 plasmid showed a higher percentage of vaccinated individuals developing a detectable cellular immune response compared to those who received the HIV DNA vaccine alone (137). Combining this vaccine regimen with *in vivo* EP dramatically amplified these results [(141); JID], showing that the inclusion of cytokine gene adjuvants with EP can improve adjuvant effects. More specifically, plasmid IL-12 and EP are capable of generating positive effects both in large animal models and in humans. Expanded studies of this important combination are in progress.

## Conclusion

The early promise of DNA vaccination had been tempered by lackluster immune responses in large animal models and humans. However, technological advances in the last decade have generated renewed interest in the improved, synthetically designed, and newly formulated DNA vaccine, especially when delivered by enhanced EP systems. Improved plasmid delivery via *in vivo* adaptive EP and the use of genetic adjuvants (in particular as plasmid-encoded IL-12) have proven to be powerful enhancers of DNA vaccines. Not only have these strategies improved immune responses in a variety of preclinical vaccination studies, but increasing evidence is suggesting that these approaches can also augment immune responses in humans. Given the various advantages of DNA vaccines – their ease of design, strong safety record, and stability, amongst others – the enhancements in immune responses in large animal models and humans is incredibly encouraging for the viability of DNA vaccines as a competitive vaccine platform. To carry these promising results further, additional research is needed on novel adjuvants, the timing of adjuvant administration, and the combination of genetic adjuvants and EP for optimal vaccination protocols. The prospects for treatment and prevention of human and animal disease by DNA vaccines are exciting, and the continual refinement of these technologies bode well for the present and future of this vaccine field.

## Conflict of Interest Statement

The laboratory of David B. Weiner has grant funding and collaborations, advising, or consulting including serving on scientific review committees for commercial entities and therefore notes possible conflicts associated with this work with Pfizer, Inovio, BMS, Virxsys, Ichor, Merck, Althea, VGXI, J&J, Aldevron, and possibly others. No writing assistance was utilized in the production of this manuscript. The other co-authors declare that the research was conducted in the absence of any commercial or financial relationships that could be construed as a potential conflict of interest.
